# A nationwide assessment of lifestyle medicine counseling: knowledge, attitudes, and confidence of Israeli senior family medicine residents

**DOI:** 10.1186/s12875-020-01261-3

**Published:** 2020-09-11

**Authors:** Lilach Malatskey, Yael Bar Zeev, Rani Polak, Adva Tzuk-Onn, Erica Frank

**Affiliations:** 1 Israeli Society of Lifestyle Medicine, Israeli Association of Family Physicians, Tel Aviv, Israel; 2 Siaal Research Center for Family Medicine and Primary Care, Department of Family Medicine, Faculty of Health Sciences, Ben-Gurion University of the Negev, POB 653, 84105 Beer-Sheva, Israel; 3 NextGenU.org, Nanoose Bay, British Columbia, Canada and Clear Lake, Washington, USA; 4 Braun School of Public Health and Community Medicine, Hebrew University of Jerusalem, Jerusalem, Israel; 5 Department of Physical Medicine & Rehabilitation, Harvard Medical School, Spaulding Rehabilitation Hospital, Boston, MA USA; 6 Center of Lifestyle Medicine, Sheba Medical Center, Tel Hashomer, Israel; 7 Faculty of Medicine, University of British Columbia, Vancouver, Canada

**Keywords:** Lifestyle medicine, Family medicine, Residents, Educational program, Lifestyle medicine course, Health behavior

## Abstract

**Background:**

Non-communicable diseases are the leading causes of death, largely due to the last century’s often-unhealthy lifestyles. Family medicine (FM) and other physicians can improve patients’ lifestyle behaviors, yet FM residency programs in Israel and other countries do not uniformly deliver lifestyle medicine (LM) training. The readiness of FM residents to counsel on lifestyle issues is not known. The purpose of this study is to assess knowledge, attitudes, and confidence levels of senior Israeli FM residents regarding LM counseling, and to evaluate the influence of LM training and personal health behaviors on residents’ LM knowledge, attitudes, and confidence.

**Methods:**

From May to June 2017, we surveyed all senior Israeli FM residents regarding their knowledge, attitudes, confidence, and personal health behaviors. We compared health behaviors, attitudes, and confidence in counselling between: 1) trained residents vs. untrained residents; 2) physically active residents vs. not physically active residents; 3) residents with a BMI < 25 vs. those with a BMI > 25; and 4) residents who eat a Mediterranean diet vs. those who do not.

**Results:**

A total of 169 senior Israeli FM residents were surveyed, and 143 completed the survey, a response rate of 84.6%. Senior FM residents said they considered LM counseling to be an integral part of their role and an effective tool by which to improve a patient’s health. Yet, their knowledge of LM and their confidence in delivering LM counseling are low. Compared with untrained residents (*n* = 84), LM-trained residents (*n* = 55) had higher knowledge scores (30.9% vs. 13.1%, *p* = 0.016) and were more confident in their ability to impact their patients’ behaviors (53.7% vs. 34.5%, *p* = 0.004). Residents’ positive personal health behaviors correlated with a higher level of confidence to provide LM counseling.

**Conclusions:**

FM physicians can play a key role in the management of patients with chronic diseases. Israeli FM residents consider counseling patients about a healthy lifestyle to be an integral part of their work, but do not feel well prepared to do so.

Dedicated LM training and resident’s personal health promotion may improve critically important levels of LM counseling and patient outcomes, and this training should therefore become a higher priority.

## Background

Non-communicable diseases (NCDs) are leading causes of death globally due, to a large extent, to unhealthy lifestyle choices of the last century [1].

In 2015, seven of the ten leading causes of death in Israel were NCDs [2]. Contrary to recommendations, an estimated 19.7% of the adult population smoked, 53.5% were overweight or obese, only 18.9% consumed at least five servings of vegetables and fruits per day, and only 35.2% routinely fulfilled physical activity recommendations [3].

More than 40% of chronic conditions could be avoided through the adoption of healthy lifestyle recommendations, according to Mokdad et al. [4], with data from the Nurses’ Health Study (1980–2014; *n* = 78,865) and the Health Professionals Follow-up Study (1986–2014, *n* = 44,354) suggesting that healthy lifestyles could substantially reduce premature mortality in U.S. adults. This would require healthier lifestyle habits such as not smoking, maintaining a body mass index (BMI) of 18.5 to 24.9 kg/m^2^, participating in at least 30 min most days of moderate to vigorous physical activity, drinking only moderate amounts of alcohol, and having a high diet quality score (upper 40%) [5].

Physicians have the ability to influence patients’ lifestyle behaviors [6–8]^.^ In a randomized controlled trial in four community-based group family medicine clinics in southeastern Missouri (adult patients, − *N* = 915), a physician’s advice was a catalyst for changes made by patients, such as quitting smoking, eating less fat, or getting more exercise, prior to receiving intervention materials on the same topic [8].

Nevertheless, most physicians do not routinely advise on lifestyle behavior changes during medical clinic visits [6, 9–11]. Healthy Israel 2020 places primary care physicians at the forefront of health promotion and preventive medicine [12]. This can be achieved by incorporating Lifestyle Medicine (LM) as part of routine clinical work and medical education programs [13, 14].

Physicians’ personal health behaviors are also related to patients’ health behaviors [15, 16]. Counseling by a physician is strongly and consistently related to the physician’s own health practices, so addressing those practices is key to increasing health promotion counseling in practice. Patients also find physicians who briefly relate that they have such healthier habits more credible and more motivating [17].

In 2015, the World Medical Association recommended [18] that national medical associations “should recognize the strong and consistent link between physicians’ and patients’ personal health practices, providing yet another critically important reason for health systems to promote physician health” and that physicians’ “workplaces should promote conditions conducive to healthy lifestyles, including access to healthy food choices, exercise, nutrition counseling and support for smoking cessation.”

A scientific statement from the American Heart Association published in 2016 recommended that all primary care residencies would benefit from incorporating lifestyle modules into their training programs [19]. The American College of Lifestyle Medicine recently launched a LM Residency Curriculum, starting its beta version application in July 2018 at four selected pilot sites [20].

The Israeli syllabus [21] for LM was developed in 2014. Since then, the Israeli Society of Lifestyle Medicine has promoted LM syllabus-based teaching in Israel. In Israel, there are five centers with Family Medicine (FM) residency programs delivering an academic diploma course of three to 4 years of weekly study days. Even though LM curricula are considered important and have been implemented in some of the FM residency programs in Israel, we know little about residents’ LM knowledge attitude nor their confidence in delivering LM counseling.

### Study aims

The aims of this study are 1) to assess the knowledge level, attitudes, and confidence of senior Israeli FM residents regarding LM counseling, and 2) to evaluate the influence of training in an LM course and the influence of personal health behaviors on residents’ knowledge, attitudes, and confidence regarding LM counseling.

## Methods

### Study design and participants

The study is a cross-sectional study.

### Participants

All FM residents are required to attend an academic diploma course, held at five different sites. All senior Israeli FM residents (those in their third or fourth years of a 4 year Israeli FM residency, *n* = 169) were included; these sites cover all geographic regions in Israel and include residents from all Israeli Health Maintenance Organizations (HMOs). LM training is usually delivered during the second year of residency or later.

### Procedure

A manual survey was provided by the researcher during one of the diploma course sessions for self-completion in one to three waves until all attended residents had completed the survey (May–June 2017). Residents were not required to provide any identifying information, and voluntary survey completion implied consent.

### Survey

Questions focused on participants’ self-reported attitudes and confidence about LM counseling, personal lifestyle behaviors, previous exposure to LM education, and LM knowledge assessment.

*Knowledge assessment* included ten questions either from FM board examinations (2010–2013) or from experts in each field. Missing answers were considered incorrect. Answering at least seven questions correctly was considered a passing level of knowledge. A score of 60% or less was considered failing the knowledge test.

*Participants’ self-reported attitudes and confidence toward LM counseling* was assessed by the Lifestyle Questionnaire. This validated questionnaire was developed to assess FM current status (internal reliability ɑ = 0.84) [22]; the questionnaire was used in a similar population in our previous study [23]. This instrument included the following: (1) thirteen items assessing attitudes regarding LM on a four-point Likert scale (1 = absolutely disagree; 4 = absolutely agree; α = 0.78), further dichotomized as agree (absolutely agree plus agree) versus disagree (absolutely disagree plus disagree), (2) nine items assessing perceived confidence regarding LM counseling on a four-point Likert scale (1 = absolutely not confident; 4 = absolutely confident; α = 0.82). Composite mean scores, a serial variable, were also measured for all attitude items combined and for all confidence items combined.

*Lifestyle behavior*s were assessed using the physician’s health questionnaire [24]. This questionnaire was developed to assess the health behavior of Israeli physicians and was subjected to internal validation by an expert panel and a convenience sample of 30 respondents. It previously had been used in an unpublished study with 4832 Israeli physicians. Topics included perceived physical activity (minutes per week aerobic activity), nutrition (days per week consuming a Mediterranean diet, which is characterized by a high intake of plant-based foods [vegetables, legumes, fruits, nuts, cereals (mainly whole grain)], olive oil as the main source of fat, moderate amounts of dairy [yoghourt and cheese], low or moderate consumption of fish and meat, and moderate consumption of wine consumed with meals [25]), processed food consuming, > = 5 servings of fruit and vegetable servings consumed/day, smoking (yes/no), sleep (average hours per night), health status (“excellent” to “bad”, on a scale of one to five), emotional stress (very little to very much, on a scale of one to five), and height and weight (to calculate body mass index (BMI). *Previous exposure to LM education* included one “yes/no” question and an estimate of hours spent on LM learning. LM syllabus-based teaching, described in our previous publication [23], is not uniformly delivered in the different LM residency teaching sites. In some sites, residents are exposed to limited LM topics. Residents may be exposed to LM materials in their HMO family department training opportunities. “Trained” residents were considered those who said they attended the LM-specific course or spent 20 h or more on LM learning.

*Sociodemographic items* were collected, including age, gender, country of birth, and country of graduation. A copy of the survey is available (in Hebrew) upon request from the authors.

### Data analysis

Data analysis was performed using SPSS Statistic Software version 23.0. Descriptive analyses of variables used counts and percentages for categorical measures and mean and standard deviation (SD) for ordinal measures.

Bivariate analysis was performed using Chi square and Fisher exact tests for categorical variables, and the Mann-Whitney U test for ordinal variables. An explorative *p* < 0.05 was considered statistically significant.

We compared health behaviors, attitudes, and confidence in counselling between trained residents (those who participated in a full LM course [23]) versus untrained residents.

We also compared attitudes and confidence between: 1) Residents who are physically active (defined as performing more than 150 min/week of exercise) and those not physically active, 2) Residents with a BMI of less than 25 versus those with a BMI of more than 25, and 3) Residents who eat a Mediterranean diet versus those who don’t. These comparisons were performed for each attitude and confidence statement separately and also for the composite scores.

Multivariate linear regression analysis was conducted to explore the association between the training and both the mean composite attitude score, and the mean composite confidence score, controlling also for age, gender, place of Medical studies, mean knowledge score, and health behaviors (reporting more than 150 min of physical activity a week, eating a Mediterranean diet, and having a BMI under 25).

### Ethics

This study was approved by the Ben-Gurion University of the Negev’s ethics committee (approval #2016–7, on February 22, 2016). The study was exempted by the ethics committee from signing informed consent forms.

## Results

### Study population characteristics

A total of 169 senior Israeli FM residents were surveyed, and 143 completed the surveys (84.6% response rate).

The participant’s average age was 36.1 years (SD 4.7) (range 29–54), and more than half were women (53.3%). In addition, 79.9% were born in Israel and almost half (48.1%) graduated from medical school in Israel.

Fifty-five participated in a LM course (i.e,” trained residents”) and 84 (“untrained residents”) did not (missing *n* = 4). No sociodemographic differences were found between “trained” and “untrained” residents (Table 1).

**Table 1:** Study population characteristics

	Trained residents (***N*** = 55)		Untrained residents (***N*** = 84)		Total (***N*** = 143)		***p*** value
	N	%	N	%	N	%	
**Age**							
**Average ± SD**	35.9 ± 4.3		36.2 ± 4.9		36.1 ± 4.7		0.680
**Range**	30–50		29–54		29–54		
	51	(mis = 4)	81	(mis = 3)	133	(mis = 10)	
**Gender**							
**Male**	24	47.1%	38	45.8%	63	46.7%	1.000
**Female**	27	52.9%	45	54.2%	72	53.3%	
	51	(mis = 4)	83	(mis = 1)	135	(mis = 8)	
**Country of birth**							
**Israel**	41	80.4%	65	79.3%	107	79.9%	0.999
**Abroad**	10	19.6%	17	20.7%	27	20.1%	
	51	(mis = 4)	82	(mis = 2)	134	(mis = 9)	
**Medical School graduation**							
**Israel**	25	50.0%	36	46.2%	62	48.1%	0.807
**Abroad**	25	50.0%	42	53.8%	67	51.9%	
	50	(mis = 5)	78	(mis = 6)	129	(mis = 14)	

### FM residents’ health behaviors

Residents reported performing an average of 115 min (SD 88.7) of physical activity per week, with an average BMI of 23.9 (SD 4.3) divided into 23 (18/1%) underweight, 57 (44.9%) normal weight, 37 (29.1%) overweight and 10 (7.9%) obese participants (missing 16).

In addition, 72.1% reported eating a Mediterranean diet at least 3 days a week and 69.8% reported eating processed foods less than twice a week. Residents reported sleeping for an average of 6.5 h per night (SD 1.2). Only 6% were current smokers; 72.1% reported mild to moderate levels of stress, and 94.3% described their general health status as mostly good.

### FM residents’ knowledge

Of the respondents, 20.1% (*n* = 28, missing 4) had a high composite knowledge score. Trained residents were significantly more likely to have a high composite knowledge score compared to untrained residents (30.9% versus 13.1%, *p* = 0.016).

### The attitude of FM residents toward their role as healthy lifestyle consultants

Most FM residents (98.6%) said they believe that LM counseling is part of their role (mean 3.83, SD 0.41) and that LM counseling is effective (82.6% agreed, mean 3.10, SD 0.69).

As shown in Table 2, compared to untrained residents, trained residents were more likely to agree that physicians have a higher impact on their patients’ behavioral changes than other providers (92.6%, mean 3.26, SD 0.65 and 81%, mean 3.00, SD 0.66, *p* < 0.01, respectively). The mean composite attitude score was significantly higher among trained residents compared to untrained residents, (2.97 (SD 0.297) and 2.85 (SD 0.232), respectively; *p* = 0.006) (Table 2). There were no statistically significant differences in mean composite attitude scores regarding participants socio-demographic characteristics (including age, sex, and country of birth), and also for the mean composite knowledge score. Israeli medical graduates had significantly higher mean composite attitude scores (*n* = 62, 2.98 [SD 0.246]), compared to participants conducting their medical studies abroad (*n* = 67, 2.84 [SD 0.268]) (*p* = 0.002).

**Table 2:** Family medicine residents’ attitudes toward their role as healthy lifestyle consultants

	LM trained residents (***N*** = 55)			LM untrained residents (***N*** = 84)			Total (***N*** = 143)			***P*** value
	Mean	STD	N	Mean	STD	N	Mean	STD	N	
Composite attitude score	2.97	0.279	55	2.85	0.232	84	2.91	0.26	143	0.006
My profession does not only include treating diseases. It includes lifestyle counseling, as well.	3.85	0.356	55	3.81	0.452	84	3.83	0.416	143	0.698
Lifestyle counseling is very effective.	3.22	0.686	55	3	0.698	83	3.1	0.698	142	0.079
Patients expect physicians to be role models in their personal health behaviors.	3.47	0.604	55	3.36	0.633	84	3.41	0.62	143	0.281
Physicians have a higher impact on their patients’ behavioral changes than other providers.	3.26	0.65	54	3	0.66	79	3.11	0.665	135	0.019
Patients can be motivated to change their behaviors, including:										
Smoking cessation	3.04	0.576	55	3.02	0.514	84	3.03	0.53	143	0.884
Losing weight	3.09	0.591	54	3	0.609	82	3.03	0.603	138	0.418
Engaging in physical activity	3.11	0.577	53	2.91	0.592	82	2.99	0.588	137	0.063
Many physicians do not guide their patients toward a healthy lifestyle because:										
They do not believe in the effectiveness of counseling.	2.55	0.741	55	2.34	0.741	82	2.43	0.75	140	0.123
Employers do not compensate for counseling.	2.65	0.731	54	2.43	0.821	81	2.53	0.794	138	0.095
Residents lack relevant knowledge.	2.62	0.828	55	2.37	0.679	81	2.47	0.745	139	0.071
It is not a part of their job.	2.09	0.701	55	2.06	0.705	83	2.09	0.702	141	0.994
It is less interesting to them.	2.53	0.663	55	2.48	0.82	82	2.51	0.763	140	0.801
They lack time.	3.31	0.639	54	3.21	0.646	81	3.26	0.645	137	0.366

Range: 1–4; 1- definitely do not agree, 4 - definitely agree. *P value for LM-trained residents versus residents not trained in LM

### FM residents’ confidence in LM consultation

Less than half (42.1%) of the residents reported that they felt confident or absolutely- confident in their ability to support patients’ lifestyle behavior changes. Trained residents reported a significantly higher proportion of feeling confident or absolutely confident, as compared to untrained residents (53.7% vs. 34.5%, *p* = 0.004).

Confidence in the ability to provide LM counselling was significantly higher in trained residents in general, especially with regard to physical activity, obesity and nutrition, as compared to untrained residents (Table 3).

**Table 3:** Family medicine residents’ assessment of confidence in healthy LM consultations

	LM trained residents (***N*** = 55)			LM un-trained residents (***N*** = 84)			Total (***N*** = 143)			***P*** value
	Mean	STD	N	Mean	STD	N	Mean	STD	N	
Composite confidence score	2.89	0.435	54	2.68	0.432	83	2.77	0.44	137	0.008
In general, it is easy to incorporate lifestyle counseling into the clinic’s daily routine.	2.67	0.777	54	2.53	0.77	83	2.59	0.769	139	0.248
I have the knowledge to promote a healthy lifestyle to my patients.	2.98	0.658	54	2.73	0.646	83	2.84	0.662	139	0.024
I can provide my patients with enough knowledge and skills to change the following behaviors:										
Cigarette smoking	2.91	0.708	54	2.73	0.7	83	2.81	0.711	139	0.154
Obesity	3.09	0.652	54	2.83	0.601	83	2.94	0.634	139	0.019
Physical inactivity	3.2	0.562	54	2.89	0.585	83	3.02	0.595	139	0.003
Sleep disturbance	2.67	0.824	54	2.64	0.636	83	2.65	0.711	139	0.812
Stress	2.78	0.718	54	2.58	0.718	83	2.65	0.719	139	0.093
Sexuality	2.41	0.714	54	2.17	0.721	81	2.28	0.725	137	0.023
Unhealthy eating	3.15	0.684	54	2.84	0.573	83	2.96	0.63	139	0.007

Range: 1–4; 1 - definitely do not agree, 4 - definitely agree

*P value for LM - trained residents versus residents not trained in LM

The mean composite confidence score for trained residents was significantly higher compared to untrained residents (2.89 [SD 0.435] and 2.68 [SD 0.432] respectively; *p* = 0.008) (Table 3). No difference was found in the mean composite confidence score for all the socio-demographic characteristics, including place of medical studies (Israel or abroad), or mean composite knowledge score. There was a weak positive correlation between the age of the participants and their mean composite confidence scores (*r* = 0.213, *p* = 0.014).

### Association between residents’ health behavior, attitudes, and confidence toward LM counselling

There was a significant difference in the mean composite attitude scores between participants who were physically active vs those reporting performing < 150 min of physical activity per week (3.04 [SD 0.294] and 2.89 [SD 0.225], respectively; *p* = 0.005); between participants with BMI < =25 vs. those with BMI > =25 (2.95 [SD 0.261[and 2.83 [SD 0.255] respectively; *p* = 0.01); and between participants reporting eating a Mediterranean diet vs. those who did not (2.94 [SD 0.282] and 2.82 [SD 0.183] respectively; *p* = 0.011).

Residents who are physically active, have a BMI<25, and ate a Mediterranean diet most days of the week had a significant higher confidence in their ability to provide patients with knowledge and skills to change their unhealthy lifestyle (Table 4).

**Table 4:** Association between residents’ health behavior, attitudes, and confidence levels with respect to LM counseling

**Physical activity**	Physical activity more than 150 min/week (*N* = 29)			Physical activity less than 150 min/week (*N* = 78)			*P* value
	Mean	STD	N	Mean	STD	N	
In my personal life, I lead a healthy lifestyle.	3.48	0.57	29	2.91	0.56	78	< 0.0001
I think patients can be persuaded to lose weight.	3.24	0.58	29	2.97	0.58	77	0.038
I think patients can be persuaded to exercise regularly.	3.21	0.57	28	2.97	0.51	77	0.041
**BMI**	BMI < 25 (*N* = 80)			BMI > 25 (*N* = 47)			*P* value
	Mean	STD	N	Mean	STD	N	
In my personal life, I lead a healthy lifestyle.	3.18	0.57	80	2.62	0.57	47	< 0.0001
I think patients can be persuaded to lose weight.	3.14	0.55	78	2.85	0.66	47	0.013
I think patients can be persuaded to exercise regularly.	3.09	0.54	78	2.85	0.67	46	0.038
I’m sure I can give patients enough knowledge and skills to improve an unhealthy diet.	3.05	0.62	78	2.79	0.66	47	0.026
**Mediterranean diet**	Eat Mediterranean diet most days of the week (*N* = 101)			Do not eat Mediterranean diet most days of the week (*N* = 39)			*P* value
	Mean	STD	N	Mean	STD	N	
In my personal life, I lead a healthy lifestyle.	3.07	0.62	101	2.69	0.61	39	0.002
Counseling for a healthy lifestyle is very effective.	3.02	0.7	100	3.33	0.66	39	0.016
In general, it is easy to include LM counseling as part of clinic activities.	2.67	0.8	101	2.37	0.63	38	0.056
I can provide my patients with enough knowledge and skills to change the following behaviors:							
Cigarette smoking	2.9	0.73	101	2.55	0.6	38	0.019
Obesity	3.01	0.66	101	2.76	0.54	38	0.044
Physical inactivity	3.1	0.59	101	2.82	0.56	38	0.012
Sleep disturbance	2.73	0.75	101	2.42	0.55	38	0.031
Unhealthy eating	3.06	0.65	101	2.71	0.52	38	0.004
I have the knowledge to promote a healthy lifestyle to my patients.	2.91	0.66	101	2.66	0.63	38	0.09

Range: 1–4; 1 - definitely do not agree, 4 - definitely agree

* *P* value for residents with healthy behaviors versus residents withhealth behaviors below recommendation levels

For the mean composite confidence score, only those reporting eating a Mediterranean diet had significantly higher confidence scores vs those reporting not eating a Mediterranean diet (2.83 [SD 0.459] and 2.60 [SD 0.348], respectively; *p* = 0.005).

Results from multivariate linear regression show that performing more than 150 min a week of physical activity (beta 0.175 [SE 0.06], 95% CI 0.055, 0.296, *p* = 0.005); eating a Mediterranean diet (beta 0.137 [SE 0.065], 95% CI 0.007, 0.267, *p* = 0.039); and completing medical school in Israel (beta 0.142 [SE 0.059], 95% CI 0.024, 0.259, *p* = 0.019) were found to be significant predictors of a higher mean composite attitude score.

Eating a Mediterranean diet (beta 0.258 [SE 0.117], 95% CI 0.024, 0.492, *p* = 0.031); completing medical school in Israel (beta − 0.293 [SE 0.105], 95% CI -0.502, − 0.084, *p* = 0.007); and the age of the participant (beta 0.022 [SE 0.011], 95% CI 0.001, 0.044, *p* = 0.039); were found to be significant predictors of a higher mean composite confidence score (Table 5).

**Table 5:** Multivariate linear regression model regarding Attitude and confidence

		OR	Sig.	95% CI	
				Lower Bound	Upper Bound
Attitude	Physical activity of more than 150 min per week	0.314	0.002	0.073	0.299
	age (years)	−0.129	0.186	−0.017	0.003
	gender (male)	0.008	0.934	−0.097	0.105
	Medical school graduation	0.316	0.002	0.062	0.265
Confidence	age (years)	0.173	0.042	0.001	0.033
	gender (male)	0.091	0.298	− 0.073	0.236
	Eating a Mediterranean diet	0.325	0.000	0.154	0.492
	Medical school graduation	−0.184	0.042	−0.322	− 0.006

## Discussion

The aims of this study were: 1) to assess the knowledge level, attitudes, and confidence of senior Israeli FM residents in regard to LM counseling, and 2) to evaluate the influence of training in an LM course and personal health behaviors on resident’s’ knowledge, attitudes, and confidence.

We delivered a nationwide survey of Israeli senior FM residents (those attending their third or fourth residency year) to assess their levels of knowledge, attitudes, and confidence with regard to LM counseling, along with the effects of LM training and personal health behaviors on those levels. Our response rate was 85%, higher than typically achieved in a physician population [26].

Our results show that senior FM residents consider LM counseling to be an integral part of their role and an effective tool by which to improve a patient’s health. Yet, their LM knowledge level and the confidence to deliver that knowledge are low.

Trained residents had a higher level of LM knowledge and were more confident in their ability to succeed and impact a patient’s behavioral changes. However, in the multivariate linear regression models, participation in the LM training was not found to be a significant predictor of either the mean composite attitude or confidence score. Personal health behaviors (such as eating a Mediterranean diet) and completing medical school in Israel were found to be significant predictors of both the mean composite attitude and confidence scores.

Personal positive health behaviors were correlated with a higher level of confidence to provide patients with knowledge and skills to change their unhealthy lifestyles.

In a study of Ohio primary care residents, scores of attitude and self-efficacy regarding obesity, nutrition, and physical activity counseling were low [27]. Another study of U.S. residents (*n* = 101) areas found that only 19% felt competent in prescribing weight-loss programs [28].

In our study, trained residents based on the Israeli LM syllabus [29] had a higher level of LM knowledge and were more confident in their ability to help patients change. Yet, in the multivariate linear regression models, LM training was not found to be a significant predictor of high confidence level. Our previous study demonstrated that immediately after a LM course, trainings improve residents’ knowledge levels and confidence in delivering LM [23].

We are aware of the low number of trained residents passing the knowledge test and their therefore appropriately limited confidence in their ability to deliver LM interventions. This may mean that more in-depth training is needed to achieve better results. The challenge is to prioritize effective LM education as an essential part of FM residency.

The LM-positive attitude of Israeli FM residents is an important finding, since it implies that there is no need to convince them of the importance of LM.

Lifestyle behaviors of Israeli FM residents are mostly healthy, which is consistent with FM physicians in the United States [27] and better than lifestyle behaviors of Israeli physicians in general [24]. FM residents see themselves as role models for their patients, and the high correlation we found between residents’ lifestyle behaviors and their perceived efficacy to impact patients demonstrates it. Prior studies have presented similar findings of a strong and consistent relationship between physicians’ and their patients’ health behaviors [15, 16, 18], and recommend investing in physicians’ health as an efficient and beneficent way to increase high-quality LM counseling and more distal patient outcomes [15, 16, 18, 30]. These findings that the residents’ personal health behaviours were correlated with their LM related attitudes and confidence, also support the need to invest in physicians’ personal health.

This study has a few limitations. First, the knowledge questionnaire was not validated and thus might not reflect actual knowledge well. Yet, it was created from a peer-reviewed FM board exam questionnaire and reviewed by experts in the field who teach in residents’ LM courses. Therefore, we believe the questions are reliable. Second, the data collected were based on self-report, including self-reported lifestyle behaviors and residents’ recollections of the amount of LM training. This is similar to other studies in this field, but we cannot rule out reporting bias around attitudes and behaviors and an incorrect recollection of the amount of LM training. Third, this study did not track LM counseling in residents’ clinics with real patients. After evaluating residents’ perceptions about LM counseling and the feasibility of LM training in Israel, our next step will be to conduct a randomized controlled trial to evaluate the impact of LM training, with a personal health promotion component, on residents’ actual counseling skills and practices. This has been shown in one four-year-long medical school intervention to be effective [30–32], but never with residents or practicing physicians.

We are in the beginning of offering widely scaled LM education programs [33]; such can be found here at the LifestyleMedicineEducation.org/curricular resources/ website. It is clear that such offerings can help developing training programs for physicians in order to enhance the health of the population.

## Conclusions

FM plays a key role in the management of patients with chronic diseases. Israeli FM residents consider consulting on healthy lifestyles to be an integral part of their work, but they are not well prepared to do so. This LM training improved these residents’ knowledge levels, but not their attitude nor their confidence to deliver LM -- but personal health behaviors improved residents’ attitude and confidence scores.

In depth dedicated LM competency-based training [33]and resident personal health promotion can improve levels of LM counseling and patient outcomes.

We recommend encouraging an expanded LM course in FM residency educational programs in Israel and abroad [33], with a significant investment to promote residents’ personal health;

Further studies are needed to examine the effect of training on patient health outcomes.

## Data Availability

Data can be made available on request from the corresponding author, following approval from the study’s approving ethics committee.

## Abbreviations

FM: Family medicine
LM: Lifestyle medicine
NCDs: Non-communicable diseases
HMOs: Health maintenance organizations
BMI: Body mass index
SD: Standard deviation
